# Prevalence of anal dysplasia and HPV genotypes in gynecology patients: The *ANGY* cross-sectional prospective clinical study protocol

**DOI:** 10.1371/journal.pone.0276438

**Published:** 2022-10-21

**Authors:** Basile Pache, Vincent Balaya, Jérôme Mathis, Martin Hübner, Roland Sahli, Mathias Cavassini, Christine Sempoux, Patrice Mathevet, Martine Jacot-Guillarmod

**Affiliations:** 1 Gynecology Department, Department Women-Mother-Child, Lausanne University Hospital (CHUV), Lausanne, Switzerland; 2 University of Lausanne, Lausanne, Switzerland; 3 Department of Gynecology and Obstetrics, Foch Hospital, Suresnes, France; 4 Gynecology and Obstetrics Department, Biel Hospital Center, Biel, Switzerland; 5 Department of Obstetrics and Gynecology, University Hospital of Berne and University of Berne, Berne, Switzerland; 6 Department of Visceral Surgery, Lausanne University Hospital (CHUV), Lausanne, Switzerland; 7 Formerly at the Institute of Microbiology, Lausanne University Hospital (CHUV), Lausanne, Switzerland; 8 Division of Infectious Diseases, Lausanne University Hospital, Lausanne, Switzerland; 9 Institute of Pathology, Lausanne University Hospital (CHUV), Lausanne, Switzerland; Teikyo University, School of Medicine, JAPAN

## Abstract

**Background:**

Human Papillomaviruses (HPV) are highly prevalent in the sexually active populations, with a significant burden in terms of health and psychological cost in all class ages. High-risk (HR) HPV genotypes are associated with anogenital dysplasia and cancers, and anal HPV-induced cancer is increasingly observed in women. The interactions of HPV genotype’s between the anus and the cervix, and the subsequent occurrence of dysplasia remains unclear. This clinical study set out to test the hypothesis that risk factors for anal HR-HPV and dysplasia may differ in women with or without cervical dysplasia or in HIV-positive women.

**Methods:**

Cervical and anal HPV genotypes and cytology testing will be performed prospectively in a cohort of women recruited in a tertiary university hospital in Switzerland. Women will be allocated to three groups: 1) normal previous cervical smear; 2) high-grade cervical dysplasia (H-SIL) at previous cervical smear; 3) HIV+, independently of previous cervical smear result. General inclusion criteria comprised the followings: Female—Age > = 18 years; Satisfactory understanding of French; No objection to HIV testing. Specific inclusion criteria are: Group 1, no past or current gynecological dysplasia and HIV negative; Group 2, Gynecological dysplasia (H-SIL) or carcinoma in situ demonstrated by histology (vulvar, vaginal or cervical) and HIV negative; Group 3: HIV-positive (regardless of viremia or CD4 count) with or without gynecological dysplasia. General exclusion criteria are: Pregnancy; History of anal dysplasia/cancer; Status after pelvic radiotherapy; Absence of anus and anal canal. Estimated prevalences of anal dysplasia are: in group 1, 1% (0–2%); in group 2, 15% (5–27%), and in group 3, 30% (19–45%). With a 10% margin error, a sample size of 120 women per group is required to reach 90% power for detecting statistical significance (unilateral α error of 5%).

**Discussion:**

The primary endpoint is the prevalence of anal and cervical dysplasia, and description of the respective HPV genotypes in each group. The results of this study could improve the standard of screening of cervical and anal dysplasia in women through evidence of concomitant presence of HPV’s and/or dysplasia in anus or cervix to support vaccination for instance. Beginning of recruitment started in September 2016. Results should be presented in end of 2022. Preliminary analysis for first 100 patients reveals that the mean age of the population is 39.6 (± 10.9) years with mean age of first sexual intercourse of 18.5 (± 3.9) years. In this cohort, 12% are vaccinated and 38% having had anal intercourse. Overall, 43% of the studied population had cervical HR-HPV in the studied population, and 53% had normal cytology. Anal LR HPV and HR HP were found in 27.6% and 38.4% of all patients respectively. Eighty percent had normal anal cytology. Groups 1,2 and 3 had a significant difference in terms of age, gestity, parity, age of first sexual intercourse, systematic use of condom, number of cervical LR HPV and HR HPV and abnormal cervical cytologies.

**Trial registration:**

The study was approved by the institutional review board—**CER-VD#2015–00200**—on the 29^th^ of June 2016 and is registered on the Swiss National Clinical Trials Portal (SNCTP), **SNCTP000002567**, Registered 29 June 2016, https://www.kofam.ch/en/snctp-portal/study/40742/

## Background

Cancer of the anal canal is rare with an estimated incidence rate of 1/100’000, being more frequently observed in women than in men [1]. This pathology has been recognized to increase in industrialized countries in both men and women for the past 3 to 4 decades [2]. The association between Human Papilloma Virus (HPV) infection and cervical cancer is well established [3]. Moreover, HPV genotypes are classified according to their oncogenic risk. The recently updated IARC classification (https://monographs.iarc.who.int/wp-content/uploads/2018/06/mono90.pdf) evaluate the HPV’s types according to their carcinogenic risks to human. In cervical lesions, IARC group 1 include HPV 16 and 18 (hereafter high risk HPV (HR-HPV), whereas potential high-risk HPV (herafter indeterminate risk HPV (IR-HPV) and low risk (LR-HPV) include others HPV types [4]. As shown in previous studies establishing the link between HR-HPV and cervical cancer, a meta-analysis of 29 cancer studies demonstrated that HR-HPV is the cause of 84% of anal cancer cases [5]. As for cervical HPV infections, anoperineal HPV infections are sexually transmitted and highly prevalent in certain risk groups, in which the incidence of anal cancer is markedly increased. These risk groups include men having sex with men (MSM) regardless of their HIV status, HIV-infected men and women, organ transplant recipients, and women with a personal history of high-grade cervical lesions [6]. The same HR-HPV (such as HPV16 or HPV18) found in cervical cancer cases, are also attributed to severe anal diseases [4]. As in the cervix, persistent anal HPV infection appears to be mandatory in the pathophysiological pathway of anal intraepithelial neoplasia and invasive cancer [7].

The prevalence of anal HPV infection is poorly documented in the general population, especially in women, because most studies target high-risk patients’ groups. While a few studies have assessed the anal prevalence of HPV in young and healthy women, studies on the prevalence of anal dysplasia in this population are lacking. However, the prevalence of anal dysplasia in patients with known gynecologic dysplasia appears to be higher than in the population without gynecologic dysplasia, in the range of 10–15% [8]. This association might be explained by the proximity of both genital and anal spheres, sex practices, or other factors.

It would be interesting to know whether women with cervical HPV infection should be monitored for anal dysplasia, since these patients might be at a higher risk for non-cervical HPV-related cancer [9]. The synchronous presence of HPV in both genital and anal area argues in favor of HPV vaccination, since it may also provide extra genital benefits. Similarly, we believe that the results of this trial will provide support for improving screening strategies for anal dysplasia, especially for women followed up in colposcopy clinics.

## Methods

### Study design–setting of the study

This study is a prospective cross-sectional, single-center, observational, comparative study which is conducted at the Lausanne University hospital (CHUV). Subjects are recruited from the policlinic and the specialized consultation of colposcopy of the Department of Gynecology and Obstetrics of the CHUV, and the HIV consultation of the CHUV. The participants are divided in three groups as follows:

- Group 1: control women.
- Group 2: women with severe gynecological dysplasia(s) (severe dysplasia and/or carcinoma in situ of the cervix (H-SIL), vagina (H-SIL) or vulva (H-SIL)).
- Group 3: HIV+ women (AIDS patients not excluded).

### Characteristics of participants

General inclusion criteria comprised the follows: Female; Age greater than or equal to 18 years; Satisfactory understanding of French; Signed informed consent; No objection to HIV testing.

General exclusion criteria are: Pregnancy; History of anal dysplasia or anal cancer; Status after pelvic radiotherapy; Absence of anus and anal canal (congenital or after surgery); Poor understanding of French.

Specificities for group 1: Patients have neither gynecological dysplasia nor a history of gynecological dysplasia. They are eligible in this group if the previous cervical smear was within the norm (Independent of HPV status). HIV serology must be negative.

Specificities for group 2: Patients in this group have gynecological dysplasia or carcinoma in situ, which may be vulvar, vaginal or cervical. Dysplasia (H-SIL) is demonstrated by cervical, vaginal or vulvar biopsy examination.

Specificities for group 3: Patients in this group are HIV+ regardless of viremia or CD4 count. They may or may not have gynecological dysplasia.

### Description of intervention

**Fig 1:**
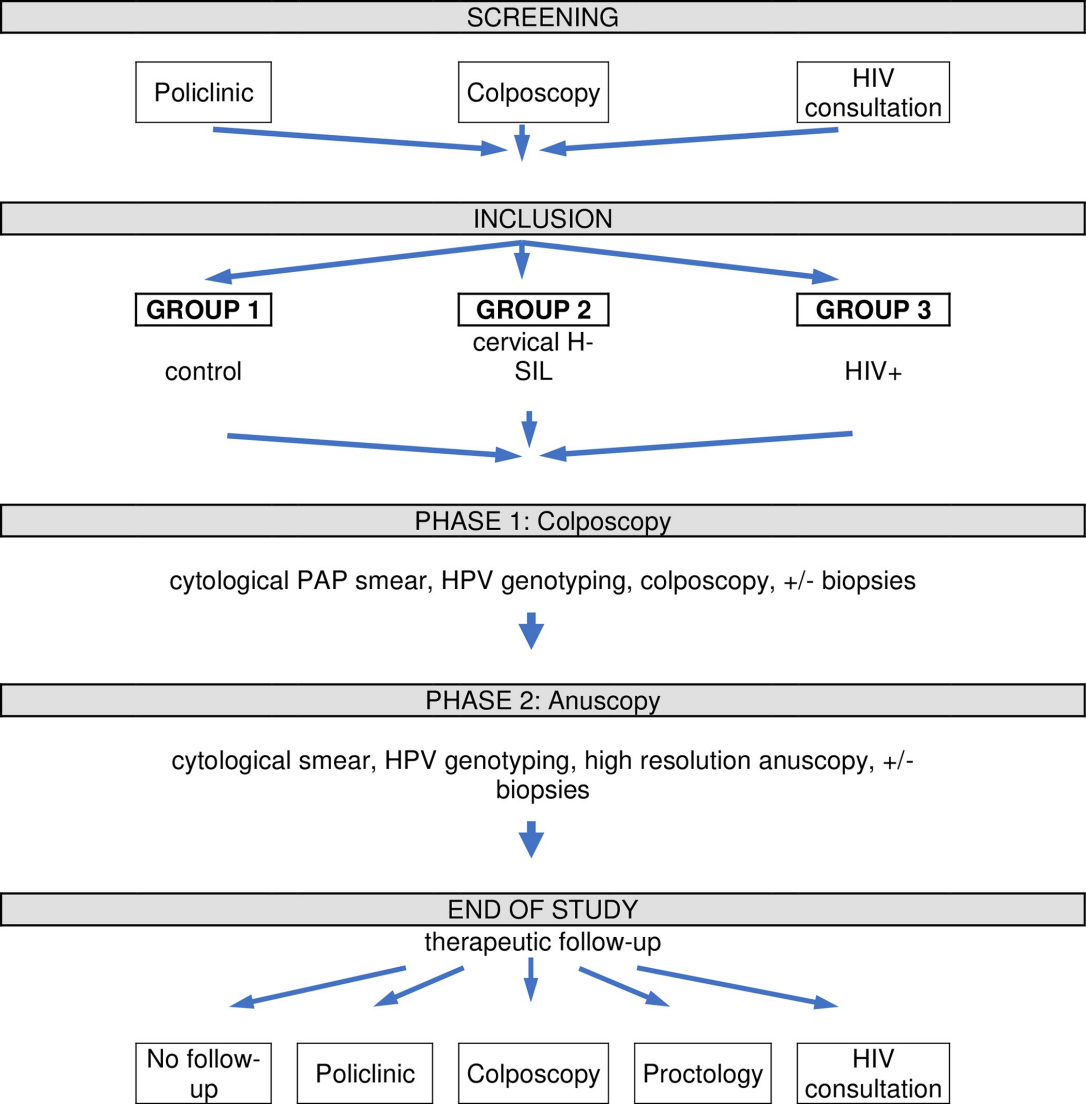
Flow chart of study protocol.

In practice, data are collected during a single consultation according to a 2-step procedure: gynecologic examination and colposcopy followed by proctologic examination and anoscopy. The Flowchart protocol is described in **Fig 1**.

Step 1 (colposcopy): This examination is performed on a gynecological chair and includes: a cervical cytology smear (for PAP-test and HPV test), a colposcopy examination with biopsies if indicated according to the international standards of care in colposcopy.

Step 2 (proctology): The examination takes place on the same examination chair but performed by the proctology team and includes: a native anal examination, a cytological smear of the anus and the anal canal (for cytology examination and HPV test), a high resolution anoscopy (HRA) with biopsies if lesions are visualized.

Blood sampling: 2 serum tubes, one for CD4 count and another for HIV testing.

Questionnaire: 14 questions are asked, through a self-completed questionnaire mainly aiming personal medical and sexual history (see the **S1 File**).

### Specificities

#### Tissue samplings

Ectocervical and endocervical sampling are done with a Plastic Cell Sampler (Deltalab, Spain) and a Cervex-Brush (Rovers Medical Devices, The Netherlands), respectively. Anal sampling is done with a Cervex-Brush (Rovers Medical Devices, The Netherlands).

After collection, cytology testing is performed on cervical and anal samples using Liquid-Based Cytology (LBC) (ThinPrep®, Hologic, USA), prior to molecular testing. Sampling is performed before any use of gel for colposcope/anoscope.

#### Cytology and histology

All cytology and histology are processed at the Institute of Pathology of CHUV and cytology results are reported according to the Bethesda 2014 classification [10].

#### HPV DNA genotyping

HPV testing is processed at the Molecular Diagnostic Laboratory of the Institute of Microbiology of CHUV. LBC residual samples are transferred from the Institute of Pathology to the Institute of Microbiology. DNA from 200 μL cell suspension is isolated with the MagNaPure 96 Total Nucleic Acids kit according to the manufacturer (Roche, Basel, Switzerland) and eluted in 100 μL elution buffer. Five μL are used for genotyping of 28 HPV genotypes (6, 11, 16, 18, 26, 31, 33, 35, 39, 40, 42–45, 51–54, 56, 58, 59, 61, 66, 68–70, 73, and 82) by the Anyplex™ II HPV28 kit according to the manufacturer (Seegene, Seoul, South Korea). HPV negative tests are valid only if their internal positive control is positive (human DNA target). Negative controls are tested in parallel throughout the procedures to monitor contaminations, as described in a previous publication [11].

#### Colposcopy and histological examination of cervix

Colposcopic evaluation is performed according to the 2011 colposcopic terminology of the International Federation for Cervical Pathology and Colposcopy [12]. Routine colposcopic examination is done with a magnifying microscope and visualization of vulva, vagina and cervix. Examination with acetic acid (5%), and subsequent application of Lugol’s iodine solution is performed. Decision to take biopsies are made upon clinical impression and physician discretion: namely within normal limits, Low-grade Squamous Intraepithelial Lesion (LSIL), High-grade

Squamous Intraepithelial Lesion (HSIL) or cancer according to the recommendations from the College of American Pathologists and the American Society for Colposcopy and Cervical Pathology [13].

#### Anoscopy and histological examination of anus

HRA is performed according to common practice evaluation [14, 15]. An anoscope is inserted and a magnifying microscope is used to examine the anal canal. Further examination with acetic acid (5%) is performed. A decision for biopsy is based on clinical impression: namely within normal limits, LSIL, HSIL or cancer.

### Statistical analysis—Sample size

The calculation of the number of patients in each group was based on a prevalence calculation. According to the current literature [16–18] the prevalence of anal dysplasia in each of the three groups are:

- Group 1 (healthy patients): 1% (0–2%)
- Group 2 (patients with gynecological dysplasia): 15% (5–27%)
- Group 3 (HIV+ patients): 30% (19–45%)

Based on these estimates and to reach a power of 90% for highlighting a significant difference at the two-sided 0.05 level, 110 patients in each group are needed between group 1 and 2 and 41 patients between group 1 and 3. By taking in account a 10% margin error, a sample size of 120 women per group is required to reach 90% power for detecting statistical significance (unilateral α error of 5%).

Qualitative variable will be expressed as n (%) and will be compared by applying the chi-squared test. Quantitative variable will be expressed as mean ± Standard deviation (SD) and will be compared by applying the ANOVA test. P values lower than 0.05 are retained as significance set. Analysis will be adjusted on the following potential confounding factors: age, ethnicity, education, smoking, anal sex, number of partners, presence of pre-existing anal lesions and age at first intercourse. Statistical analysis will be carried out using XLstat Biomed software (AddInsoft V19.4, Paris, France).

### Ethics approval and consent to participate

The study was approved by the local review board “*Commission cantonal d’éthique de la recherche sur l’être humain*” (“State commission for human ethical research”)—**CER-VD#2015–00200**—on the 29^th^ of June 2016 and was registered at the Swiss National Clinical Trials Portal (SNCTP) under the reference: **SNCTP000002567**, https://www.kofam.ch/en/snctp-portal/study/40742/

## Results

Preliminary analysis of first 100 patients is reported hereafter. Demographic of patients are shown in **Table 1**. Mean age was 39.6 years +/-10.9 [22–78], Gestity 2.1+/-2.1 [0 – 16], Parity 1.4+/- 1.2 [0 – 4], with higher education being secondary school for 26.3% of patients, and university for 24.2%. Mean age for first sexual intercourse 18.5+/-3.9 years old [13 – 41]. 38% had sexual intercourse. 82% had not been vaccinated against HPV. 84% had not used condom for their regular sexual intercourse. 24% had only one partner, when 19.8% had more than 10 sexual partners. 73.7% were non-smokers.

**Table 1:** Patient characteristics.

Predictive variable	Overall population N = 100	
	n Mean ± SD	[%] [range]
Demographic and Anamnesis		
**Age [years]**		
**Mean**	39.6 ± 10.9	[22–78]
**Gestity**		
**Mean**	2.1 ± 2.1	[0–16]
**Parity**		
**Mean**	1.4 ± 1.2	[0–4]
**Education**		
**Secondary school**	25	26.3
**Apprenticeship**	28	29.5
**High school**	11	11.6
**University of applied sciences**	8	8.4
**University**	23	24.2
**Not specified**	5	
**Age of first sexual intercourse**		
**Mean**	18.5 ± 3.9	[13–41]
**Anal Intercourse**		
**Yes**	38	38.0
**No**	62	62.0
**HPV vaccinated**		
**Yes**	12	12.8
**No**	82	87.2
**Not specified**	6	
**Condom used**		
**Yes**	13	13.4
**No**	84	86.6
**Not specified**	3	
**Number of sexual partners**		
**0**	2	2.1
**1**	23	24.0
**2–4**	28	29.2
**5–10**	24	25.0
**> 10**	19	19.8
**Not specified**	4	
**Smoking**		
**Yes**	25	26.3
**No**	70	73.7
**Not specified**	5	

PCR genotyping, cytology and pathology in the overall population, respectively for the cervix and the anus are presented in **Table 2A and 2B**. Cervical LR HPV was found in 21% of all patients, and Cervical HR HPV in 43%. 53% had normal cytology, 14.1% LSIL, 4.3% HSIL, 18.5% ASCUS and 9.8% ASC-H. 37% of patients had cervical biopsies, of which 2.7% were normal, 13.5% were LSIL and 83.8% HSIL. Anal LR HPV was found in 27.6% of all patients, and anal HR HPV in 38.4%. 80% had normal cytology, 5.9% LSIL, 5.9% HSIL, 5.9% ASCUS and 2.4% ASC-H. 6% of patients had anal biopsies, of which 16.7% were normal, 66.7% were LSIL and 16.7% HSIL.

**Table 2:** Overall population: PCR genotyping, cytology, pathology. A) Cervical analysis B) Anal analysis.

Predictive variable	Overall population N = 100	
	n Mean ± SD	[%] [range]
PCR genotyping, cytology, pathology		
**Cervical analysis**		
**Cervical HPV PCR**		
**Cervical HPV LR**		
**Yes**	21	21.0
**No**	79	79.0
**Cervical HPV HR**		
**Yes**	43	43.0
**No**	57	57.0
**Cervical pathology**		
**Cervical cytology**		
**Normal**	49	53.3
**LSIL**	13	14.1
**HSIL**	4	4.3
**ASCUS**	17	18.5
**ASC-H**	9	9.8
**Not specified**	8	
**Cervical biopsy performed**		
**Yes**	37	37.0
**No**	63	63.0
**Cervical biopsy**		
**Normal**	1/37	2.7
**LSIL**	5/37	13.5
**HSIL**	31/37	83.8
**Anal analysis**		
**Anal HPV PCR**		
**Anal HPV LR**		
**Yes**	24	27.6
**No**	63	72.4
**Not specified**	13	
**Anal HPV HR**		
**Yes**	33	38.4
**No**	53	61.6
**Not specified**	14	
**Anal pathology**		
**Anal cytology**		
**Normal**	68	80.0
**LSIL**	5	5.9
**HSIL**	5	5.9
**ASCUS**	5	5.9
**ASC-H**	2	2.4
**Not specified**	15	
**Anal biopsy**		
**Yes**	6	6.0
**No**	94	94.0
**Anal histology**		
**Normal**	1/6	16.7
**LSIL**	4/6	66.7
**HSIL**	1/6	16.7

**Table 3** displays sub-groups univariate analysis of factors associated with cervical HPV-HR. Groups 1,2 and 3 were significantly different in terms of age, gestity, parity, age of first sexual intercourse, systematic use of condom, number of cervical LR HPV and HR HPV and abnormal cervical cytologies. There were no differences in terms of anal intercourse, HPV vaccination, number of previous sexual partners, smoking status, anal LR HPV and HR HPV and anal cytologies.

**Table 3:** Sub-groups univariate analysis of factors associated with cervical HPV-HR.

Predictive variable	Group 1 Control N = 36		Group 2 HSIL-gyn N = 41		Group 3 HIV+N = 23		*P*
	n Mean ± SD	[%] [range]	n Mean ± SD	[%] [range]	n Mean ± SD	[%] [range]	
**Demographic and Anamnesis**							
**Age [years]**							
**Mean**	42.1 ± 11.7	[22–78]	34.6 ± 9.1	[22–51]	44.7 ± 9.0	[24–57]	***0*.*0002***
**Gestity**							
**Mean**	2.8 ± 2.7	[0–16]	1.4 ± 1.2	[0–4]	2.3 ± 1.9	[0–7]	***0*.*01***
**Parity**							
**Mean**	1.9 ± 1.2	[0–4]	0.9 ± 1.0	[0–3]	1.4 ± 1.3	[0–4]	***0*.*001***
**Education**							
**Secondary school**	8	23.5	7	17.5	10	47.6	***0*.*06***
**Apprenticeship**	10	29.4	11	27.5	7	33.3	
**High school**	7	20.6	3	7.5	1	4.8	
**University of applied sciences**	2	5.9	6	15.0	0	0.0	
**University**	7	20.6	13	32.5	3	14.3	
**Not specified**	2		1		2		
**Age of first sexual intercourse**							
**Mean**	20.1 ± 3.8	[13–30]	17.1 ± 4.3	[14–41]	17.5 ± 2.6	[13–22]	***0*.*01***
**Anal Intercourse**							
**Yes**	10	27.8	19	46.3	4	39.1	*0*.*24*
**No**	26	72.2	22	53.7	19	60.9	
**HPV vaccinated**							
**Yes**	5	14.3	6	15.8	1	4.8	*0*.*45*
**No**	30	85.7	32	84.2	20	95.2	
**Not specified**	1		3		2		
**Condom used**							
**Yes**	4	11.4	1	2.5	8	36.4	***0*.*001***
**No**	31	88.6	39	97.5	14	63.6	
**Not specified**	1		1		2		
**Number of sexual partners**							
**0**	0	0.0	0	0.0	2	9.1	*0*.*17*
**1**	11	33.3	8	19.5	4	18.2	
**2–4**	10	30.3	12	29.3	6	27.3	
**5–10**	5	15.2	14	34.1	5	22.7	
**> 10**	7	21.2	7	17.1	5	22.7	
**Not specified**	3				1		
**Smoking**							
**Yes**	7	21.2	10	25.6	8	34.8	*0*.*52*
**No**	26	78.8	29	74.4	15	65.2	
**Not specified**	3		2				
**PCR genotyping, cytology, pathology**							
**Cervical HPV PCR**							
**Cervical HPV LR**							
**Yes**	1	2.8	16	39.0	4	17.4	***0*.*0004***
**No**	35	97.2	25	61.0	19	82.6	
**Cervical HPV HR**							
**Yes**	5	13.9	29	70.7	9	39.1	***<0*.*0001***
**No**	31	86.1	12	29.3	14	60.9	
**Cervical pathology**							
**Cervical cytology**							
**Normal**	25	71.4	13	33.3	11	61.1	***0*.*02***
**LSIL**	1	2.9	10	25.6	2	11.1	
**HSIL**	0	0.0	4	10.3	0	0.0	
**ASCUS**	7	20.0	7	17.9	3	16.7	
**ASC-H**	2	5.7	5	12.8	2	11.1	
**Not specified**	1		2		5		
**Cervical biopsy performed**							
**Yes**	1	2.8	32	78.0	4	17.4	***<0*.*0001***
**No**	35	97.2	9	22.0	19	82.6	
**Cervical biopsy**							
**Normal**	1/1	100.0	0/32	0.0	0/4	0.0	***<0*.*0001***
**LSIL**	0/1	0.0	2/32	6.3	3/4	75.0	
**HSIL**	0/1	0.0	30/32	93.8	1/4	25.0	
**Anal HPV PCR**							
**Anal HPV LR**							
**Yes**	5	15.2	11	30.6	8	44.4	*0*.*07*
**No**	28	84.8	25	69.4	10	55.6	
**Not specified**	3		5		5		
**Anal HPV HR**							
**Yes**	8	24.2	17	48.6	8	44.4	*0*.*10*
**No**	25	75.8	18	51.4	10	55.6	
**Not specified**	3		6		5		
**Anal pathology**							
**Anal cytology**							
**Normal**	29	90.6	25	71.4	14	77.8	*0*.*36*
**LSIL**	0	0.0	3	8.6	2	11.1	
**HSIL**	2	6.3	2	5.7	1	5.6	
**ASCUS**	1	3.1	4	11.4	0	0.0	
**ASC-H**	0	0.0	1	2.9	1	5.6	
**Not specified**	4		6		5		
**Anal biopsy**							
**Yes**	1	2.8	2	4.9	3	13.0	*0*.*25*
**No**	35	97.2	39	95.1	20	87.0	
**Anal histology**							
**Normal**	0/1	0.0	1/2	50.0	0/3	0.0	*0*.*99*
**LSIL**	1/1	100.0	1/2	50.0	2/3	66.7	
**HSIL**	0/1	0.0	0/2	0.0	1/3	33.3	

## Discussion

Anal dysplasia is the precursor of anal cancer but is not subject to systematic screening despite its growing incidence in the last decade. By analogy to cervical cancer screening, systematic screening for anal dysplasia would be the most sensitive option for anal cancer prevention [19]. Considering the prevalence of anal cancer, which remains much lower than that of cervical cancer, this option would not be acceptable neither for the patients nor in terms of public health costs. Because the number of people to screen on a regular basis, without any specific risk factors identified, would be too high for diagnosing anal dysplasia and cancer. Targeting risk groups is probably a solution that will greatly increase the sensitivity of screening at the cost of a decrease in specificity that we hope will be as small as possible.

The identification of certain risk groups of patients such as those with HIV or severe gynecological dysplasia and the calculation of their prevalence within these groups will probably allow us to argue in this direction, as they can be confounding factors. These patients could benefit from screening for anal dysplasia and hopefully reduce the number of anal canal cancers. Association of tobacco use and anal dysplasia through HPV prevalence will be studied. Anal sex, probably through a mechanism of micro-lesions of the anal mucosa, is most likely a risk factor even if the current data are unclear. Our data may show an association between the presence of anal dysplasia and anal sex. If so, it would justify that patients should be informed of this risk during the consultations about sexuality. The counseling provided in consultation could thus be individualized according to the reported sexual behaviors and target the indications for anal screening, taking into account the distribution of the different HPV genotypes in the genital and anal spheres.

The preliminary analysis of first 100 included patients gives interesting and promising insights, although those data should be carefully considered, as the power for significant results has not been met yet.

The present study has some limitations and inherent bias, beyond its cross-sectional design that does only enhance an overview of the situation and does not follow patients in time. The resources allocated to a cross sectional study from a single center limits the maximal number of patients that we can include. This may induce a limited representativity of the population, since the percentage of cervical HR HPV (43%) does not reflect the general population. Nevertheless, group 1 (no abnormal prior Papp smear) was created to ensure a sample size that could be representing the average population consulting in a day-to-day clinic of GYN/OBS in a tertiary hospital. The number of patients included does also limit the power HPV genotype’s sub-group analysis. A population selection bias is possible since anogenital HPV infection could also be managed in clinics and private practice and not only in tertiary center. Recall bias are possible, as the disease examined is slowly evolving through time and accuracy of recall regarding prior exposures can vary. The duration of the study will depend on the number of patients recruited in each of the three groups. We do not expect one group to be faster to complete than another, since all three groups of patients are recruited from three dedicated clinics in an academic center with a large caseload. Regarding the proportion of patients recruited from the total screened during the consultation, as there is no additional time during the consultation for the study and some patients do not speak/understand enough French, we might face some delay in recruitment.

The study of the different HPVs in the gynecological and anal areas will allow us to compare HPV carriage at both sites and to evaluate if the same genotypes, in our population, are associated with dysplasia whether they are found at the gynecological or anal level. If HPV 16 and 18 are involved in an increased prevalence of anal dysplasia, we will have an additional argument in favor of the HPV vaccination program in young women.

## Supporting information

S1 File Questionnaire for the participants of the ANGY study. (DOCX)

## Abbreviations

HIV: Human Immunodeficiency Virus
HPV: Human Papilloma Virus
MSM: men having sex with men
HR: HPV: high risk HPV
LR: HPV: low risk HPV
IR: HPV: indeterminate risk HPV
HRA: high resolution anoscopy
