# Supplementary material for: Prevalence of anal dysplasia and HPV genotypes in gynecology patients: The ANGY cross-sectional prospective clinical study protocol
Source: PLoS One. 2022 Oct 21;17(10):e0276438. doi: 10.1371/journal.pone.0276438 (PMC9586359; doi:10.1371/journal.pone.0276438)
Supplement: S1 File — (DOCX) [file pone.0276438.s001.docx]

**Supplementary File 1**

**Questionnaire for the participants of the *ANGY study***

1. Have you ever been pregnant? (Y/N) If yes, how many times? (number)

1. Have you had children? (Y/N) If yes, how many children do you have? (number)

2. Schooling and studies: Secondary school, Apprenticeship, Gymnasium/high school, HES, University

3. Are you taking medication for cancer or because of an organ transplant? (Y/N)

4. Are you a carrier of the AIDS virus (HIV)? (Y/N/dont' know)

5. Do you smoke cigarettes? (Y/N) If yes: number of cigarettes/day and for how many years (number).

6. How many sexual partners have you had? 0, 1, 2-5, 5-10, >10

7. At what age did you have your first sexual intercourse? (year)

8. Do you always have sex with a condom? (Y/N)

9. Have you ever had anal sex (sodomy)? (Y/N) If yes, in the past 6 months? (Y/N)

10. Have you had any anal or anal canal disease? (Y/N) If yes: Anal fistula, Anal fissure, Hemorrhoids, Other.

11. Have you been vaccinated against the HPV virus? (Y/N) If yes, which vaccine? How many injections?

12. Are you taking birth control (Y/N) If yes, which one?

13. Have you ever had an abnormal annual Pap smear? (Y/N)

14. Have you ever heard of anal cancer? (Y/N)
